# Emergence and clonal dissemination of KPC-2- and NDM-1-coharboring *Citrobacter freundii* in China with an IncR plasmid

**DOI:** 10.1128/spectrum.01953-24

**Published:** 2024-12-19

**Authors:** Juanjuan Zhou, Minghui Zhen, Kaijie Gao, Lu Xu, Dongyu Zhang, Junmei Yang

**Affiliations:** 1 Department of Clinical Laboratory, Children’s Hospital Affiliated to Zhengzhou University, Henan Children’s Hospital, Zhengzhou Children’s Hospital, Zhengzhou Key Laboratory of Children’s Infection and Immunity, Zhengzhou, China; ICON plc, Health Economics and Epidemiology, London, United Kingdom

**Keywords:** *Citrobacter freundii*, KPC-2-NDM-1-CRCFs, dissemination, comparative genomic analysis

## Abstract

The rise of carbapenem-resistant *Enterobacteriaceae* coharboring KPC-2 and NDM-1 poses a significant public health threat. KPC-2-NDM-1-*Citrobacter freundii* is rarely reported in clinical settings. In this study, we report the largest cohort of eight KPC-2-NDM-1-*C. freundii* isolated from children with urinary tract infections. Comprehensive characterization, including antimicrobial susceptibility testing, plasmid conjugation, whole-genome sequencing, and comparative genomics, was conducted for these clinical strains. Antimicrobial susceptibility testing indicated that all strains were resistant to meropenem [minimal inhibitory concentration (MICs) ≥ 8 µg/mL] and imipenem (MICs > 8 µg/mL). Genotyping and comparative genomics analyses identified the eight KPC-2-NDM-1- *C. freundii* belonging to ST523, exhibiting a close relationship characterized by 45 single nucleotide polymorphisms. Whole-genome sequencing and plasmid analysis confirmed the presence of *bla*_KPC-2_ and *bla*_NDM-1_ on an IncR plasmid named pC275-2 with 46,050 bp. The *bla*_NDM-1_ was integrated within the *bla*_NDM-1_ related region, which includes ΔIS*Aba125*, *bla*_NDM-1_, *ble-MBL*, *trpF*, *dsbD*, and IS*CR1*, while the *bla*_KPC-2_ gene was located on a novel non-Tn*4401* genetic element. The *bla*_KPC-2_ genetic architectures containing *bla*_KPC-2_ differed from classical structures, underscoring the ongoing evolution of these genetic elements.

**IMPORTANCE:**

Our study described the largest cohort to date of eight ST523 KPC-2-NDM-1-*C. freundii* isolated from children with urinary tract infections. The cooccurrence of KPC-2, a serine *β*-lactamases, and NDM-1, a metallo-β-lactamase on an IncR plasmid pC275-2 from a clinical carbapenem-resistant *C*. *freundii*. The conserved insertion structure mediated the *bla*_NDM-1_, and the propagation of *bla*_KPC-2_ gene with a new genetic background using IncR plasmid in clinical settings promotes the emergence of superbugs necessitating vigilant monitoring. Our research detected that ST523 KPC-2- NDM-1-*C. freundii* were disseminated in a children’s hospital. The potential spread of an IncR plasmid within the hospital raises concerns about the pandemic potential of this clone that produces two carbapenemases: KPC-2 and NDM-1. Further investigations will be necessary to control and prevent the spread of KPC-2-NDM-1-*C. freundii*s.

## INTRODUCTION

*Citrobacter freundii*, a Gram-negative bacterium belonging to the *Enterobacteriaceae* family, is commonly associated with various human infections, including urinary tract infections, respiratory tract infections, bloodstream infections, and neonatal meningitis (1–3). The emergence of carbapenem-resistant *C. freundii* (CRCF) is a significant concern due to its association with high in-hospital mortality (4, 5). CRCF has been linked to the presence of class A serine *β*-lactamase KPC and metallo-*β*-lactamases such as NDM, VIM, and IMP (3, 6, 7). Recent challenges in clinical treatment have arisen from the emergence of carbapenem-resistant *Klebsiella pneumoniae* (CRKP) coharboring KPC-2 and NDM-1, which has impacted the effectiveness of cephalosporins, carbapenems and *β*-lactamase enzyme inhibitors, including ceftazidime-avibactam, meropenem-vaborbactam, and imipenem-relebactam (8, 9). Concerningly, instances of KPC-2 and NDM-1 coharboring have been detected in various *Enterobacterales*, including *Serratia marcescens*, *Enterobacter hormoechei*, *Raoultella* spp., and *C. freundii* (10–13). While KPC-2 and NDM-1 coharboring CRCF (KPC-2-NDM-1-CRCF) remain relatively uncommon, existing studies have reported such cases from adult patient specimens and medical waste in China (13, 14). Moreover, cases involving KPC-2-NDM-1-CRCF in children are scarce. Notably, previous studies often presented single CRCF strain harboring *bla*_KPC-2_ and *bla*_NDM-1_ on two separate plasmids or lacked comprehensive clinical and microbiological characteristics, limiting the holistic understanding of these double-positive CRCFs (13, 14). Addressing these critical gaps is essential given the potential for “super-antimicrobial resistance” to exacerbate the public health threat posed by superbugs.

The ongoing laboratory-based surveillance of carbapenem-resistant *Enterobacteriaceae* (CRE) in children since 2017 led to the detection of eight cases of urinary tract infections caused by KPC-2-NDM-1-CRCF strains. To our knowledge, this study presents the largest number of KPC-2-NDM-1-CRCF strains, offering a unique perspective on addressing the aforementioned questions. This study highlights the emergence and outbreak potential of KPC-2-NDM-1-CRCFs in a children’s hospital, and emphasizes the urgent need for effective measures to control the spread of these multidrug-resistant strains, especially among pediatric patients.

## RESULTS

### Isolation and identification of KPC-2-NDM-1-CRCF strains

Between June 2020 and January 2021, eight nonduplicate KPC-2-NDM-1-CRCFs (C202, C224, C228, C236, C275, C284, C285, and C2177) were isolated from urine specimens of eight pediatric patients, ranging in age from 8 months to 9 years, who were diagnosed with urinary infection in the urology ward of a children’s hospital in Zhengzhou, China (Table 1). All eight patients recovered from their urological diseases.

**TABLE 1:** Clinical characteristics of children with *bla_KPC-2_* and *bla_NDM-1_ -coharboring* CRCF strains^*a*^

Patients	C202	C224	C228	C236	C275	C284	C285	C2177
Age	9m	1y5m	7y	6y11m	8m	9y	10m22d	4y
Gender	Male	Female	Male	Male	Male	Male	Male	Male
Ward	Urology	Urology	Urology	Urology	Urology	Urology	Urology	Urology
Infection	Urinary tract	Urinary tract	Urinary tract	Urinary tract	Urinary tract	Urinary tract	Urinary tract	Urinary tract
Surgery	Ureteral stent placement	Ureteral stent placement	Ureteral stent placement	Ureteral stent placement	Ureteral stent placement	Ureteral stent placement	Ureteral stent placement	Ureteral stent placement
Temperature (*T*_max_) (°C)	36.4	38.5	38.5	36.5	38.6	36.5	36.8	36.5
Length of hospitalization	10	16	8	12	17	20	11	12
Therapeutic antimicrobial usage	CAZ	SCF	SCF	SCF	CAZ	SCF	SCF	CAZ
Outcome	Recovered	Recovered	Recovered	Recovered	Recovered	Recovered	Recovered	Recovered

^*a*^ CAZ, ceftazidime; SCF, cefoperazone-sulbactam.

### Antimicrobial susceptibility and genotyping of KPC-2-NDM-1-CRCFs

Antimicrobial susceptibility testing revealed that KPC-2-NDM-1-CRCFs were resistant to all *β*-lactams and *β*-lactam/*β*-lactamase inhibitor combinations including ceftazidime (CAZ), cefotaxime (CTX), cefepime (FEP), aztreonam (AZM), amoxicillin-clavunic acid (AMC), piperacillin-tazobactam (TZP), imipenem (IMP), and meropenem (MEM). However, they remained susceptible to ciprofloxacin (CIP), colistin (COL), and tigecycline (TGC) (Table 2). Only C284 was resistant to trimethoprim-sulfamethoxazole (SXT) and other strains were susceptible to SXT. Imipenem-3-aminobenzeneboronic acid/EDTA double disk synergy test results indicated the production of both metallo- and serine-carbapenemase by these KPC-2-NDM-1-CRCFs. PCR analysis confirmed the presence of *bla*_KPC-2_ and *bla*_NDM-1_ in all eight KPC-2-NDM-1-CRCFs.

**TABLE 2:** Antimicrobial susceptibility testing of KPC-2-NDM-1-CRCFs: MIC values (μg/mL) and CLSI susceptibility interpretation^*a*^

Strains	Antimicrobial																							
	AMC		TZP		CAZ		CTX		FEP		AZM		IMP		MEM		CIP		SXT		TGC		COL	
	MIC	R/S	MIC	R/S	MIC	R/S	MIC	R/S	MIC	R/S	MIC	R/S	MIC	R/S	MIC	R/S	MIC	R/S	MIC	R/S	MIC	R/S	MIC	R/S
C202	>16/8	R	>64/4	R	>16	R	>32	R	>16	R	>16	R	>8	R	>8	R	≤0.5	S	≤0.5/9.5	S	≤0.5	S	1	S
C224	>32/16	R	>64/4	R	>16	R	>32	R	>16	R	>16	R	>8	R	8	R	≤0.5	S	1/19	S	1	S	1	S
C228	>16	R	>64/4	R	>16	R	>32	R	>16	R	>16	R	>8	R	>8	R	0.5	S	1/19	S	≤0.5	S	1	S
C236	>16/8	R	>64/4	R	>16	R	>32	R	>16	R	>16	R	>8	R	>8	R	≤0.5	S	≤0.5/9.5	S	≤0.5	S	1	S
C284	>32	R	>64/4	R	>16	R	>32	R	>16	R	>16	R	>8	R	>8	R	≤0.5	S	>4/76	R	1	S	1	S
C285	>32/16	R	>64/4	R	>16	R	>32	R	>16	R	>16	R	>8	R	>8	R	≤0.5	S	≤0.5/9.5	S	1	S	0.5	S
C2177	>32/16	R	>64/4	R	>16	R	>32	R	>16	R	>16	R	>8	R	>8	R	≤0.5	S	≤0.5/9.5	S	≤0.5	S	1	S
C275	>16/8	R	>64/4	R	>16	R	>32	R	>16	R	>16	R	>8	R	>8	R	≤0.5	S	≤0.5/9.5	S	≤0.5	S	1	S

^*a*^ MIC, minimun inhibitory concentration; AMC, amoxicillin-clavunic acid; TZP, piperacillin-tazobactam; CAZ, ceftazidime; CTX, cefotaxime; FEP, cefepime; AZM, aztreonam; IMP, imipenem; MEM, meropenem; CIP, ciprofloxacin; SXT, trimethoprim-sulfamethoxazole; TGC, tigecycline; COL, colistin.

### Genomics features of KPC-2-NDM-1-CRCF

The genomic analysis of the CRCF C275, as detailed in Table 3, revealed a circular chromosome of 5,013,530 bp with a GC content of 51.7% and three plasmids: pC275-1 (81,275 bp), pC275-2 (46,050 bp), and pC275-3 (4,619 bp). In this study, pC275-2 carried a single plasmid replicon, IncR, while both pC275-1 and pC275-3 were classified as untype able plasmids. Analysis for acquired resistance genes identified the presence of the *bla*_CMY-116_ gene encoding resistance to *β*-lactams on the chromosome. Additionally, the pC275-2 was found to carry several resistance genes conferring resistance to *β*-lactams (*bla*_KPC-2_, *bla*_NDM-1_), bleomycin (*ble*MBL), and sulfonamide (*sul1*). However, no antimicrobial resistance genes were identified in the pC275-1 and pC275-3 plasmids.

**TABLE 3:** Genomic features of *Citrobacter freundii* C275

Feature	Chromosome	Plasmid 1	Plasmid 2	Plasmid 3
Total number of bases (bp)	5,013,530	81,275	46,050	4,619
G + C content (%)	51.7	53.3	55.8	51.1
No. of protein-coding sequences	4774	98	51	8
No. of rRNA genes	25	0	0	0
No. of tRNA genes	85	0	0	0
Plasmid replicon type	−^*a*^	−	lncR	−
Resistance genes	CMY-116, dfrA3		KPC-2, NDM-1, MBL, sull	

^*a*^ "-", No plasmid replicon.

### Whole-genome sequencing analysis and characterization of plasmid sequence carrying *bla_KPC-2_* and *bla*_NDM-1_

**Fig 1:**
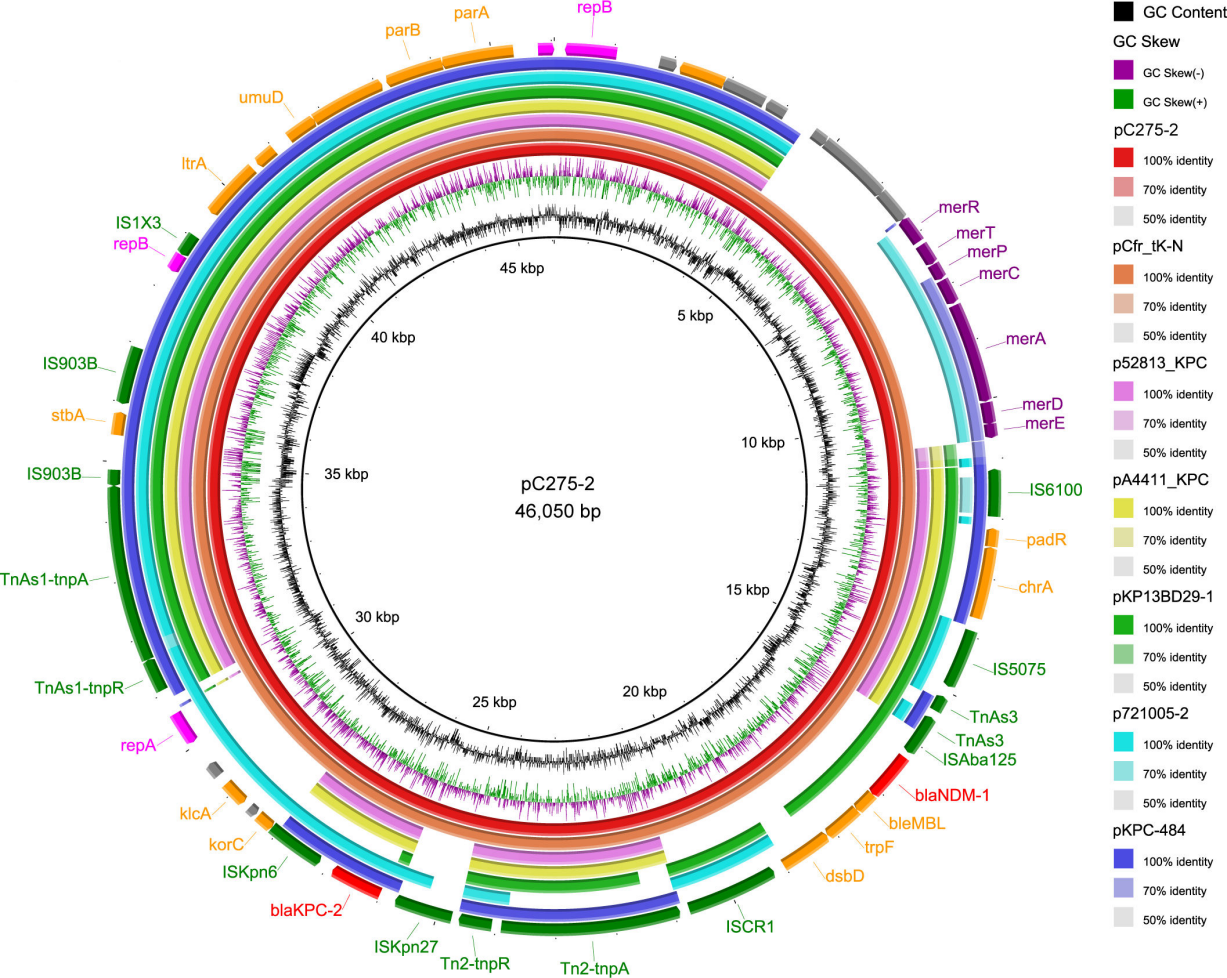
Comparative genome analysis of pC275-2 with pCfr_tK-N, p52813_KPC, pA4411_KPC, pKP13D29-1, p721005-2, and pKPC-484. Circles inside to outside denote the GC content, GC skew and the ORFs in both DNA strands. Antimicrobial resistance genes are highlighted in red. Virulence factors are highlighted in purple.

**Fig 2:**
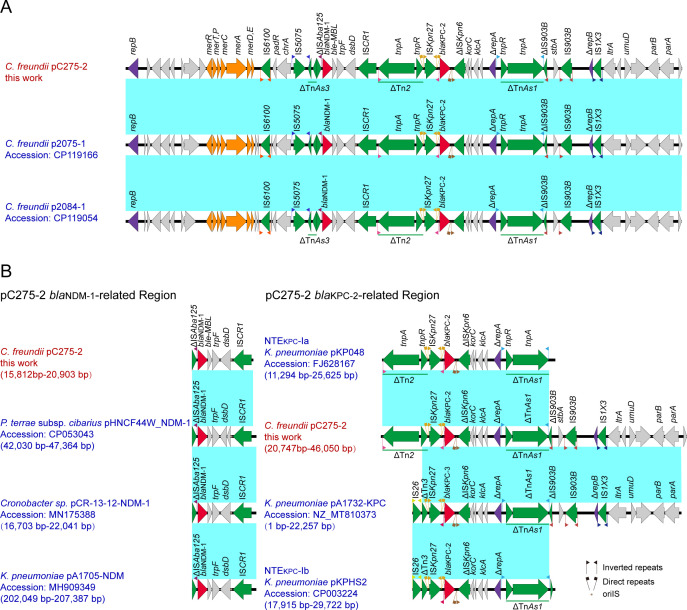
(**A**) Colinear genome alignment of pC275-2, p2075-1 (CP119166), and p2084-1 (CP119054). (**B**) Linear alignment of pC275-2 *bla*_NDM-1_-related region or pC275-2 *bla_KPC-2_*-related region.

The 46 kb IncR length plasmid carrying *bla*_KPC-2_ and *bla*_NDM-1_ was assembled entirely and designated as pC275-2 (Fig. 1). The pC275-2 exhibited 100% query coverage and 99% nucleotide identity with pCfr_tK-N (CP119168) from *C. freundii*. It also demonstrated the following similarities with various plasmids from *K. pneumoniae*: 66% query coverage with p52813_KPC, 66% query coverage with pA4411_KPC, 70% query coverage with pKP13D29-1, 73% query coverage with p721005-2, and 69% query coverage with pKPC-484, all maintaining a nucleotide identity of 99%. The linear comparison revealed that the region of pC275-2 carrying resistance genes was highly similar with p2084-1 and p2075-1 (CP119054 and CP119166, respectively) (100% query coverage and >99% nucleotide identity) from the *C. freundii* strain (Fig. 2A). Regarding species origin, except for the above three sequences, other similar sequences were mainly derived from *K. pneumoniae* (MG764550.1).

The pC275-2 plasmid carries both *bla*_NDM-1_ and *bla*_KPC-2_ carbapenem resistance genes. The flanking structures of these two genes are in close proximity and contain multiple insertions or transposons, including IS*6100*, IS*5075*, ΔTn*As3*, ΔIS*Aba125,* IS*CR1*, ΔTn*2*, ISK*pn27*, ΔISK*pn6*, ΔTn*As1*, ΔIS*903B*, IS*903B*, and IS*1X3*. Two 6-base-pair direct repeats (DR, TATAGG) sequences were adjacent to the right inverted repeat sequence (IRL) and left inverted repeat sequence (IRR) of the ISK*pn27* (Fig. 2; Tables S1 and S2). However, *bla*_NDM-1_ and *bla*_KPC-2_ are carried by different genetic constructs and do not belong to the same whole (Fig. 2B). The *bla*_NDM-1_-related region in pC275-2 consists of ΔIS*Aba125*, *bla*_NDM-1_, *ble-MBL*, *trpF dsbD* and IS*CR1*, resembling similar structures found in other bacteria, such as *Proteus terrae* subsp. cibarius plasmid pHNCF44W_NDM-1 (CP053043), *Cronobacter* sp. plasmid pCR-13-12-NDM-1 (MN175388), *Providencia* sp. 17090510-03 (CP042861) and *K. pneumoniae* plasmid pA1705-NDM (MH909349) with high degree of coverage and similarity (>99% query coverage and >99.90% identity) (Fig. 2B; Table S3).

A BLAST search against the NCBI nucleotide database revealed that the *bla*_KPC-2_-related region of pC275-2 exhibited 86.0% query coverage and 99.8% identity to pA1732-KPC (NZ_MT810373). Except for the fact that pA1732-KPC carries the KPC gene *bla*_KPC-3_, this sequence is highly similar to the *bla*_KPC-2_ related region of pC275-2, extending from IS*Kpn27* upstream to the *parA* gene (Fig. 2B; Table S4). Further analysis indicated that the *bla*_KPC-2_-related region of pC275-2 is similar to NTE_KPC_-Ia proposed by Chen et al. (15). This region is located within a transposable structure that spans from ΔTn*2* to ΔTn*As1* and includes ΔTn*2*, IS*Kpn27*, *bla*_KPC-2_, ΔIS*Kpn6*, *korC*, *Orf*, *klcA*, *orf*, Δ*repA*, and ΔTn*As1*.

Repeated conjugative transfer experiments with pC275-2 were unsuccessful, due to the absence of a type IV secretion system (T4SS) or T4 coupling protein (T4CP). However, the presence oriT and relaxase genes suggest potential transfer facilitated by other binding transfer elements. In contrast, pC275-1 appears to be a conjugative plasmid, equipped with oriT, relaxase, T4CP coupling protein, and T4SS secretion system, while pC275-3 is presumed to be non-mobile.

### Comparative genomic analysis of KPC-2-NDM-1-CRCFs

The number of single nucleotide polymorphisms (SNPs) among the eight KPC-2-NDM-1-CRCFs was 45, suggesting probable nosocomial dissemination of the same clone. Chromosomes similarities were observed among C202, C224, C228, C236, C284, C285, and C2177 strains which were highly similar to C275, with the exception of C275 carrying *bla*_CMY-116_. All seven KPC-2-NDM-1-CRCFs contained plasmids similar to those found in C275, designated pC275-1 and pC275-2. These strains belonged to sequence type (ST) ST523. Furthermore, an investigation of the virulence determinants revealed that all strains carried fimbrial-related genes such as *fim*, *csg*, *flh*, *fli*, *flg*, and *che*.

**Fig 3:**
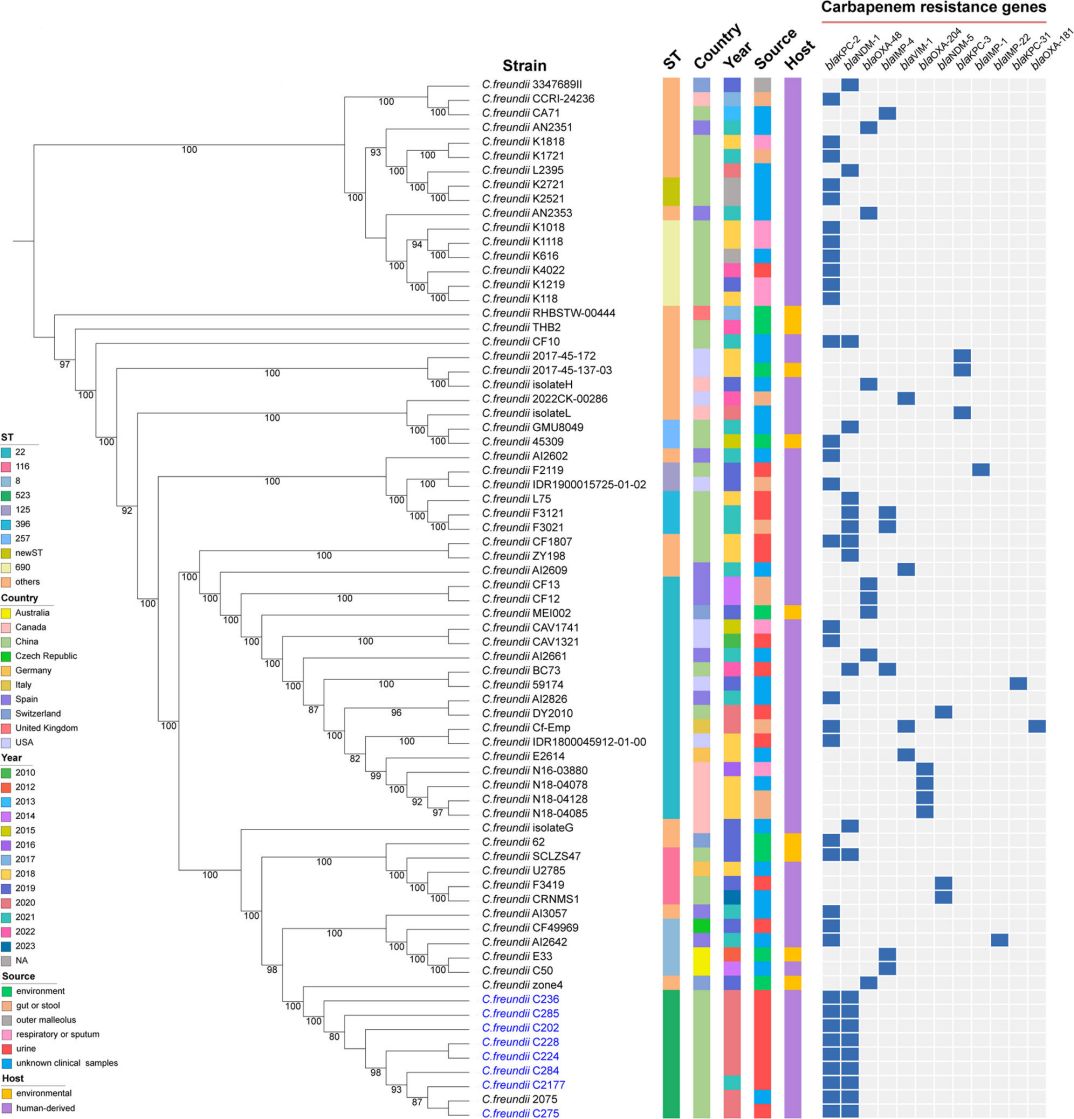
Comparative genome analysis of eight KPC-2-NDM-1-CRCFs (C275, C202, C224, C228, C236, C284, C285, and C2177) and other CRCF strains based on SNPs. The ST types, locations, collection dates, isolation sources, hosts of strains, and carbapenem resistance genes are shown.

Although CRCF genomes have been previously reported (16), few studies have linked the genetic information of multiple CRCFs to explore their evolutionary relationships and internal genome structure. To address this gap, we conducted a genomic comparison of CRCFs. These strains were detected in various specimen types including urine, sputum, stool, gut, swab, sink drain, rectal swab, and roots from homo sapiens and the environment (Fig. 3; Table S5). Simultaneously, the detection spanned across countries, such as Australia, Canada, China, Czech Republic, Germany, Italy, Spain, Switzerland, the United Kingdom, and the United States from 2010 to 2023, indicating widespread and rapid emergence of CRCFs worldwide. The main resistance gene of CRCF was *bla*_KPC_, followed by *bla*_NDM_, *bla*_OXA_, *bla*_IMP_, and *bla*_VIM_.

*C. freundii* isolate G, 62, SCLZS47, U2785, F3419, CRNMS1, E33, C50, zone4 and the eight CRCF strains (C275, C202, C224, C228, C236, C284, C285, and C2177) exhibited genetic relatedness (Fig. 3; Table S5). These eight CRCFs clustered with *C. freundii* 2075, with all nine strains being from urine samples in Henan. Further genomic analysis revealed that all of these strains contained *bla*_KPC_ and *bla*_NDM_, and harbored the same plasmid replicon IncR.

## DISCUSSION

The rapid global emergence of CRE (17), particularly those harboring KPC-2 and NDM-1 carbapenemases, poses a substantial threat to healthcare systems (18). While the detection of KPC- and NDM-producing *Enterobacteriaceae* is widespread, the coharboring of KPC-2 and NDM-1 in CRCF remains infrequently reported. This study presents a comprehensive analysis of eight KPC-2-NDM-1-CRCFs, revealing their clonal outbreak and emphasizing the critical need for infection control measures. All eight KPC-2-NDM-1-CRCFs were collected from urine samples and identified within 7 months in the same ward, indicating a clonal outbreak according to phylogenetic tree analysis and whole-genome sequencing (WGS). The potential dissemination through the transmission of an IncR plasmid within the hospital raises concerns about the pandemic potential of this dual carbapenemase-producing clone. Therefore, our findings highlight the necessity of infection control and prevention of the spread of KPC-2-NDM-1-CRCFs.

All eight KPC-2-NDM-1-CRCFs belonged to ST523. It is worth noting that the *bla*_KPC-2_ and *bla*_NDM-1_ in this study are located on an IncR plasmid, which was different from that reported in previous studies where *bla*_KPC-2_ and *bla*_NDM-1_ are situated on two separate plasmids (13, 19). This suggests that the IncR plasmid to *C. freundii* may contribute to the pandemic clone of this KPC-2-NDM-1-CRCF.

Here, we found eight CRCFs from patients with urinary infection in China, and the pC275-2 co-harbored *bla*_KPC-2_ and *bla*_NDM-1_ IncR plasmid native to the area. To our knowledge, only three *bla*_KPC-2_ and *bla*_NDM-1_ coharboring IncR plasmids have been found in *C. freundii* (strains 2075, 2084, and 2085) in China (20), which indicates that regional dissemination of such pathogens might be due to *bla*_KPC-2_ and *bla*_NDM-1_ coharboring IncR plasmid. The pC275-2 displayed 100% query coverage and 99% nucleotide identity with p2084-1 and p2075-1, indicating that these plasmids are indeed clones of pC275-2. This suggests a potential for horizontal transfer of pC275-2, an essential finding in understanding the dissemination of this plasmid within the bacterial population. While pC275-2 could not undergo autonomous transfer due to necessary T4SS and T4CP, it could be transferred with the aid of other conjugative elements. The IncR plasmid p2075-1 of *C. freundii* 2075 coharbored *bla*_KPC-2_ and *bla*_NDM-1_ could be transferred to *K. pneumoniae* KP54 with mediation by IS*6100* via transposition, with another conjugative IncFII plasmid included in the same *C. freundii* strain (20).

Plasmid pC275-2 simultaneously carries two carbapenem-resistant genes, *bla*_NDM-1_ and *bla*_KPC-2_, with their flanking structures closely adjacent to each other, along with numerous insertion sequences or transposons. The genetic surroundings of pC275-2 *bla*_NDM-1_ and *bla*_KPC-2_-related regions are separate insertion structures rather than a single entity. We observed that the *bla*_NDM-1_-related regions were found upstream and downstream of the *bla*_NDM-1_ genetic context of pC275-2. Interestingly, the conserved structure sequence is consistent with the upstream and downstream of *bla*_NDM-1_ genetic context of pHNCF44W_NDM-1 (CP053043), pCR-13-12-NDM-1 (MN175388), and pA1705-NDM (MH909349), as so as the chromosome of *Providencia rettgeri* strain Pr-15-2-50 (CP039844), which means that this gene cluster structure may play a pivotal role in extensive horizontal *bla*_NDM-1_ gene transmission. Furthermore, ΔIS*Aba125* and IS*CR1* are present on both sides of the conserved structure sequence, hinting that similar structures identified in certain bacterial chromosomes might facilitate the transposition of the *bla*_NDM-1_ gene from the plasmid to the chromosome.

The *bla*_KPC-2_ gene was located on a non-Tn*4401* element with the structure of the *bla*_KPC-2_-related regions. Compared to the classical NTE_KPC_-Ia structure in pKP048 (FJ628167), additional ΔIS*903B*, *stbA*, *IS903B*, Δ*repB*, and IS*1X3* were identified downstream of *bla*_KPC-2_ in the genetic context of pC275-2. This genetic arrangement also exhibited significant similarity to the NTE_KPC_-Ib structure in pKPHS2 (CP003224). In addition, our analysis revealed that the genetic context of *bla*_KPC-3_ in plasmid pA1732-KPC (NZ_MT810373) shared 86.0% query coverage and 99.8% identity with *bla*_KPC-2_ in pC275-2. However, the primary distinction lies in the upstream region of KPC, which contains different genes (ΔTn*2* and IS*26*). What is more, the *bla*_KPC-3_ flanking structure of pA1732-KPC is more closely related to NTE_KPC_-Ib. These findings indicate that the *bla*_KPC-2_ gene was located on a novel non-Tn4401 element in this study. Various genetic organizations containing *bla*_KPC-2_ have been identified beyond the conventional Tn*4401* since 2009 (21), with documented mutations surrounding *bla*_KPC_ (22). The novel element can be classified as the group I NTE element proposed by Chen et al. (15). Unlike the classic NTE_KPC_-Ia structure lacking ΔIS*903B*, *stbA*, *IS903B*, Δ*repB*, and IS*1X3* downstream of *bla*_KPC_, these elements are in the new structure, implying its origin from the recombination and evolution of the NTE_KPC_-Ia structure in pKP048.

In conclusion, our study described the largest cohort to date of 8 KPC-2- NDM-1-CRCFs strains from children with urinary tract infections. We characterized a *bla*_NDM-1_- and *bla*_KPC-2_-coharboring IncR plasmid pC275-2. The *bla*_NDM-1_-related region is shared among plasmids and chromosomes of various bacteria potentially facilitating the transposition of *bla*_NDM-1_ from the plasmid to the chromosome. The *bla*_KPC-2_ gene was surrounded by novel genetic contexts, which differ somewhat from previous reports. The dissemination of two crucial resistance genes with the novel genetic contexts via IncR plasmid in clinical settings will contribute to the rise of superbugs, which increases the need for vigilance in the clinic.

## MATERIALS AND METHODS

### Species identification and confirmation of carbapenemase production

The laboratory-based surveillance of CRE was previously outlined (18). Species identification was initially performed using Bruker Biotyper MALDI-TOF MS (Bruker Daltonik GmbH, Bremen, Germany) and further confirmed by 16s rRNA gene sequencing. The phenotypic detection of carbapenemase was carried out using the Imipenem-3-aminobenzeneboronic acid/EDTA double disk synergy test. Carbapenemase genes (KPC, NDM, OXA, VIM, and IMP) were identified via PCR and DNA sequencing as detailed previously (18). The study protocol adhered to the Declaration of Helsinki and was approved by the Zhengzhou University Ethics Committee. Bacteria samples were sourced from children’s normal medical activities. This study has no impact on the patients, and was anonymized, no patient informed consent statement was required

### Antimicrobial susceptibility testing

Each *C. freundii* isolate underwent resistance testing using the broth microdilution method, with *E. coli* ATCC 25922 as a control. The interpretation of results and quality control followed the 2023 CLSI breakpoints for various antibiotics, including AMC, TZP, CTX, CAZ, FEP, AZM, MEM, IMP, CIP, and SXT. TGC and COL (MICs were evaluated according to the European Committee for Antimicrobial Susceptibility Testing criteria (23).

### Conjugation assay and plasmid sequencing

Conjugation experiments were conducted to assess plasmid transferability using rifampicin-resistant *E. coli* 600 as the recipient strain. The conjugants were selected on Mueller-Hinton agar (OXOID, Hampshire, United Kingdom) supplemented with 2 mg/L of meropenem and 200 mg/L of rifampicin (Meilunbio, Dalian, China). The transconjugants were detected by MALDI-TOF MS (Bruker Daltonik GmbH, Bremen, Germany). Antimicrobial susceptibility testing and *bla*_KPC-2_ and *bla*_NDM-1_ were performed. The conjugation elements were identified by the oriTfinder tool (https://bioinfo-mml.sjtu.edu.cn/oriTfinder/).

### WGS and bioinformatics analysis

The genomic DNA of all eight CRCFs was extracted using the SDS method. DNA quality was measured using a TBS-380 fluorometer (Turner BioSystems Inc., Sunnyvale, CA, USA) and NanoDrop 2000 spectrophotometer (Thermo Fisher Scientific, USA). Genome sequencing of eight CRCFs was performed using a paired-end library with an average insert size of 350 bp via Illumina NovaSeq PE150 (Illumina, San Diego, CA, USA), followed by *de novo* assembled by SPAdes 3.10.0 (24). For further analysis, the whole genome of *C. freundii* C275 was sequenced using the PacBio Sequel platform (Pacific Biosciences, CA, USA) and Illumina NovaSeq PE150 (Illumina, San Diego, CA, USA). The genome assembly process utilized SOAP denovo 2.04 (25), SPAdes 3.10.0 (24), and Abyss 1.3.7 (26) software, applying various K-mer configurations to generate initial results. These results were then merged using CISA 1.3 (27). Gaps were addressed using gapclose software 1.12, low-depth reads were excluded, and fragments under 500 bp were eliminated to complete the assembly for gene prediction. The genome was annotated using RAST version 2.0 (https://rast.nmpdr.org) and BLAST (https://blast.ncbi.nlm.nih.gov/Blast.cgi). Antibiotic resistance genes and plasmid replicons were identified using online tools (https://www.genomicepidemiology.org/). The ST was determined by MLST (https://cge.food.dtu.dk/services/MLST/). The IS Finder database was scanned for transposon and IS elements and integrons (https://www-is.biotoul.fr/). Plasmid comparisons were performed using BLAST Ring Image Generator (BRIG) (http://brig.sourceforge.net) and Easyfig tools (http://mjsull.github.io/Easyfig).

### Comparative genomic analysis of KPC-2-NDM-1-CRCF

Genome sequences 65 CRCFs were downloaded from Pathogen Detection (https://www.ncbi.nlm.nih.gov/pathogens/) and analyzed alongside the eight CRCFs using kSNP3 (https://doi.org/10.1093/bioinformatics/btv271). A phylogenetic tree was constructed using PhyML based on concatenated, qualified SNPs, with the whole genome of C275 as the reference. The tree was used to visualize and annotate using ITOL (https://itol.embl.de).

## Data Availability

The genome sequencing data are publicly available at NCBI GenBank with the following accession numbers: C275 chromosome, CP161897; p275-1, CP161898; p275-2, CP161899; p275-3, CP161900.

## SUPPLEMENTAL MATERIAL

Table S1 spectrum.01953-24-s0001.doc The inverse repeat sequences of the region of blaKPC-2 and blaNDM-1 in pC275-2.

Table S2 spectrum.01953-24-s0002.doc The direct repeat sequences of the region of blaKPC-2 and blaNDM-1 in pC275-2.

Table S3 spectrum.01953-24-s0003.doc Genetic context of blaNDM-1 on pC275-2，pHNCF44W_NDM-1，pCR-13-12-NDM-1 and pA1705-NDM.

Table S4 spectrum.01953-24-s0004.doc Genetic context of blaKPC-2 on pC275-2，pKP048，pA1732-KPC and pKPHS2.

Table S5 spectrum.01953-24-s0005.doc Characteristics of all carbapenem-resistant Citrobacter freundiis included in comparative genomic analysis.

ASM does not own the copyrights to Supplemental Material that may be linked to, or accessed through, an article. The authors have granted ASM a non-exclusive, world-wide license to publish the Supplemental Material files. Please contact the corresponding author directly for reuse.

## References

[1] Liu LH, Wang NY, Wu AYJ, Lin CC, Lee C-M, Liu CP. 2018. Citrobacter freundii bacteremia: risk factors of mortality and prevalence of resistance genes. J Microbiol Immunol Infect 51:565–572. doi:10.1016/j.jmii.2016.08.016 28711438

[2] Samonis G, Karageorgopoulos DE, Kofteridis DP, Matthaiou DK, Sidiropoulou V, Maraki S, Falagas ME. 2009. Citrobacter infections in a general hospital: characteristics and outcomes. Eur J Clin Microbiol Infect Dis 28:61–68. doi:10.1007/s10096-008-0598-z 18682995

[3] Yang L, Li P, Liang B, Hu X, Li J, Xie J, Yang C, Hao R, Wang L, Jia L, Li P, Qiu S, Song H. 2018. Multidrug-resistant Citrobacter freundii ST139 co-producing NDM-1 and CMY-152 from China. Sci Rep 8:10653. doi:10.1038/s41598-018-28879-9 30006537 PMC6045649

[4] Babiker A, Evans DR, Griffith MP, McElheny CL, Hassan M, Clarke LG, Mettus RT, Harrison LH, Doi Y, Shields RK, Van Tyne D. 2020. Clinical and genomic epidemiology of carbapenem-nonsusceptible Citrobacter spp. at a tertiary health care center over 2 decades. J Clin Microbiol 58:e00275-20. doi:10.1128/JCM.00275-20 32554477 PMC7448640

[5] Liu L, Chen D, Liu L, Lan R, Hao S, Jin W, Sun H, Wang Y, Liang Y, Xu J. 2018. Genetic diversity, multidrug, and virulence of Citrobacter freundii from diarrheal patients and healthy individuals. Front Cell Infect Microbiol 8:233. doi:10.3389/fcimb.2018.00233 30050870 PMC6052900

[6] Ouyang J, Sun F, Zhou D, Feng J, Zhan Z, Xiong Z, Yang B, Liu Z, Li T, Tong Y, Xia P. 2018. Comparative genomics of five different resistance plasmids coexisting in a clinical multi-drug resistant Citrobacter freundii isolate. Infect Drug Resist 11:1447–1460. doi:10.2147/IDR.S165818 30254476 PMC6140695

[7] Gaibani P, Ambretti S, Farruggia P, Bua G, Berlingeri A, Tamburini MV, Cordovana M, Guerra L, Mazzetti M, Roncarati G, Tenace C, Moro ML, Gagliotti C, Landini MP, Sambri V. 2013. Outbreak of Citrobacter freundii carrying VIM-1 in an Italian Hospital, identified during the carbapenemases screening actions, June 2012. Int J Infect Dis 17:e714–e717. doi:10.1016/j.ijid.2013.02.007 23528638

[8] Bitar I, Caltagirone M, Villa L, Mattioni Marchetti V, Nucleo E, Sarti M, Migliavacca R, Carattoli A. 2019. Interplay among IncA and bla_KPC_-carrying plasmids in Citrobacter freundii. Antimicrob Agents Chemother 63:e02609-18. doi:10.1128/AAC.02609-18 30858205 PMC6496092

[9] Gao H, Liu Y, Wang R, Wang Q, Jin L, Wang H. 2020. The transferability and evolution of NDM-1 and KPC-2 co-producing Klebsiella pneumoniae from clinical settings. EBioMedicine 51:102599. doi:10.1016/j.ebiom.2019.102599 31911273 PMC6948161

[10] Ymaña B, Luque N, Pons MJ, Ruiz J. 2022. KPC-2-NDM-1-producing Serratia marcescens: first description in Peru. New Microbes New Infect 49–50:101051. doi:10.1016/j.nmni.2022.101051 PMC9709192 36466011

[11] Pereira PS, Borghi M, Albano RM, Lopes JCO, Silveira MC, Marques EA, Oliveira JCR, Asensi MD, Carvalho-Assef APD. 2015. Coproduction of NDM-1 and KPC-2 in Enterobacter hormaechei from Brazil. Microb Drug Resist 21:234–236. doi:10.1089/mdr.2014.0171 25473727

[12] Zou H, Berglund B, Wang S, Zhou Z, Gu C, Zhao L, Meng C, Li X. 2022. Emergence of bla_NDM-1_, bla_NDM-5_, bla_KPC-2_ and bla_IMP-4_ carrying plasmids in Raoultella spp. Environ Pollut 306:119437. doi:10.1016/j.envpol.2022.119437 35537555

[13] Li Y, Fang C, Qiu Y, Dai X, Zhang L. 2022. Genomic characterization of a carbapenem-resistant Citrobacter freundii co-carrying bla_KPC-2_ and bla_NDM-1_. J Glob Antimicrob Resist 29:289–292. doi:10.1016/j.jgar.2022.04.014 35489677

[14] Huang J, Zhao J, Yi M, Yuan Y, Xia P, Yang B, Liao J, Dang Z, Xia Y. 2023. Emergence of tigecycline and carbapenem-resistant Citrobacter freundii co-carrying tmexCD1-toprJ1, bla_KPC-2_, and bla_NDM-1_ from a sepsis patient. Infect Drug Resist Volume 16:5855–5868. doi:10.2147/IDR.S426148 PMC10492580 37692469

[15] Chen L, Mathema B, Chavda KD, DeLeo FR, Bonomo RA, Kreiswirth BN. 2014. Carbapenemase-producing Klebsiella pneumoniae: molecular and genetic decoding. Trends Microbiol 22:686–696. doi:10.1016/j.tim.2014.09.003 25304194 PMC4365952

[16] Sheppard AE, Stoesser N, Wilson DJ, Sebra R, Kasarskis A, Anson LW, Giess A, Pankhurst LJ, Vaughan A, Grim CJ, Cox HL, Yeh AJ, Sifri CD, Walker AS, Peto TE, Crook DW, Mathers AJ, Modernising Medical Microbiology (MMM) Informatics Group. 2016. Nested Russian doll-like genetic mobility drives rapid dissemination of the carbapenem resistance gene bla_KPC_. Antimicrob Agents Chemother 60:3767–3778. doi:10.1128/AAC.00464-16 27067320 PMC4879409

[17] Ding L, Shen S, Chen J, Tian Z, Shi Q, Han R, Guo Y, Hu F. 2023. Klebsiella pneumoniae carbapenemase variants: the new threat to global public health. Clin Microbiol Rev 36:e0000823. doi:10.1128/cmr.00008-23 37937997 PMC10732083

[18] Zhou J, Yang J, Hu F, Gao K, Sun J, Yang J. 2020. Clinical and molecular epidemiologic characteristics of ceftazidime/avibactam-resistant carbapenem- resistant Klebsiella pneumoniae in a neonatal intensive care unit in China. Infect Drug Resist Volume 13:2571–2578. doi:10.2147/IDR.S256922 PMC7394509 32801794

[19] Qiao J, Chen Y, Ge H, Xu H, Guo X, Liu R, Li C, Chen R, Gou J, Chen M, Zheng B. 2023. Coexistence of bla_IMP-4_, bla_NDM-1_ and bla_OXA-1_ in bla_KPC-2_-producing Citrobacter freundii of clinical origin in China. Front Microbiol 14. doi:10.3389/fmicb.2023.1074612 PMC10291173 37378293

[20] Zhang F, Li Z, Liu X, Hu Y, Zhao J, Zhang Y, Fan Y, Lei Z, Yang X, Li Z, Li C, Wu Y, Lu B. 2023. Carbapenem-resistant Citrobacter freundii harboring bla_KPC-2_ and bla_NDM-1_: a study on their transferability and potential dissemination via generating a transferrable hybrid plasmid mediated by IS6100. Front Microbiol 14. doi:10.3389/fmicb.2023.1239538 PMC10469622 37664119

[21] Shen P, Wei Z, Jiang Y, Du X, Ji S, Yu Y, Li L. 2009. Novel genetic environment of the carbapenem-hydrolyzing beta-lactamase KPC-2 among Enterobacteriaceae in China. Antimicrob Agents Chemother 53:4333–4338. doi:10.1128/AAC.00260-09 19620332 PMC2764158

[22] Huang QS, Liao W, Xiong Z, Li D, Du FL, Xiang TX, Wei D, Wan LG, Liu Y, Zhang W. 2021. Prevalence of the NTEKPC-I on IncF plasmids among hypervirulent Klebsiella pneumoniae isolates in Jiangxi Province, South China. Front Microbiol 12:622280. doi:10.3389/fmicb.2021.622280B2 34234750 PMC8256152

[23] Wayne PA. 2021. Clinical and laboratory standards institute. Performance standards for antimicrobial susceptibility testing. 31st ed. Clinical and Laboratory Standards Institute, Wayne, PA, USA. 10.1128/JCM.00213-21 PMC8601225 34550809

[24] Bankevich A, Nurk S, Antipov D, Gurevich AA, Dvorkin M, Kulikov AS, Lesin VM, Nikolenko SI, Pham S, Prjibelski AD, Pyshkin AV, Sirotkin AV, Vyahhi N, Tesler G, Alekseyev MA, Pevzner PA. 2012. SPAdes: a new genome assembly algorithm and its applications to single-cell sequencing. J Comput Biol 19:455–477. doi:10.1089/cmb.2012.0021 22506599 PMC3342519

[25] Li R, Zhu H, Ruan J, Qian W, Fang X, Shi Z, Li Y, Li S, Shan G, Kristiansen K, Li S, Yang H, Wang J, Wang J. 2010. De novo assembly of human genomes with massively parallel short read sequencing. Genome Res 20:265–272. doi:10.1101/gr.097261.109 20019144 PMC2813482

[26] Simpson JT, Wong K, Jackman SD, Schein JE, Jones SJM, Birol I. 2009. ABySS: a parallel assembler for short read sequence data. Genome Res 19:1117–1123. doi:10.1101/gr.089532.108 19251739 PMC2694472

[27] Lin S-H, Liao Y-C. 2013. CISA: contig integrator for sequence assembly of bacterial genomes. PLoS One 8:e60843. doi:10.1371/journal.pone.0060843 23556006 PMC3610655

