# Supplementary material for: Emergence and clonal dissemination of KPC-2- and NDM-1-coharboring Citrobacter freundii in China with an IncR plasmid
Source: Microbiol Spectr. 2024 Dec 19;13(2):e01953-24. doi: 10.1128/spectrum.01953-24 (PMC11792461; doi:10.1128/spectrum.01953-24)
Supplement: Table S1 — The inverse repeat sequences of the region of blaKPC-2 and blaNDM-1 in pC275-2. [file spectrum.01953-24-s0001.doc]

Table S1 The inverse repeat sequences of the region of *bla*_KPC-2_ and *bla*_NDM-1_ in pC275-2

| **MGEs** | **IRs** | **Start** | **End** | **Sequence (5'→3')** |
| --- | --- | --- | --- | --- |
| IS*6100* | IRL | 11947 | 11996 | GGCTCTGTTGCAAAAATCGTGAAGCTTGAGCATGCTTGGCGGAGATTGGA |
|  | IRR | 11117 | 11166 | GGCTCTGTTGCAAAGATTGGCGGCAGTCAGAGGTAGGCTGTCGCTCTGCG |
| IS*5075* | IRL | 13844 | 13893 | TAATGAGATGGTCACTCCCTCCTTCCCAGTACTATGCTGAGGACAGGCTT |
|  | IRR | 15125 | 15170 | TCTATGGTCACTCCCGTTTTTGCAACACCGATTTTGACGACAAGTT |
|  | IRR | 16159 | 16208 | GGCGAATTCAAACATGAGGTGCGACAGTTTCAAAAGCCATATGATAATCA |
| IS*CR1* | oriIS | 18973 | 18993 | GGGTATAGGAAGTATAAACCA |
| Tn2 | IRL | 25896 | 25933 | GGGGTCTGACGCTCAGTGGAACGAAAACTCACGTTAAGAAGTCATTTTTC |
|  | IRR | 20871 | 20908 | GGGGTCTGACGCTCAGTGGAACGAAAACTCACGTTAAGCAACGTTTTCTA |
| ISKpn27 | IRL | 24648 | 24697 | TGTCTGGACTCGTGGGATCATGTACCCATGCGTAGCTGGCCGCTCTTCAA |
|  | IRR | 25678 | 25727 | TGTCAAGACCCGGCTGGTTATACACGCGTTTCCTGAACAATTCAGGGCGT |
|  | IRR | 26971 | 27020 | GGAGTCTGCGCGGCAGAGCCGTGTGACCGGTTTTCTGTAGAGCACTGACG |
| TnAs1 | IRL | 30676 | 30725 | GGGGAACCGCAGAATTCGGAAAAAATCGTACGCTAAGCTAAGGAAGGTC |
|  | IRR | 34582 | 34631 | GGGGAGCCCGCAGAATTCGGAAAAAATCGTACGCTAAGGTTTTCCGGGCA |
| IS903B | IRL | 34847 | 34896 | GGCTTTGTTGAATAAATCGAACTTTTGCTGAGTTGAAGGATCAGATCACG |
|  | IRR | 35916 | 35965 | GGCTTTGTTGAATAAATCAGATTTCGGGTAAGTCTCCCCCGTAGCGGGTT |
| IS1X3 | IRL | 39365 | 39414 | GGATAATGGTGCCAACTTACTGATTTAGTGTACGATGGTGTCTTTGAGGT |
|  | IRR | 38646 | 38695 | GGTAATGACTCCAACTTACTGATAGTGTTTTATGTTCAGATAATGCCCGA |
