# Supplementary material for: Emergence and clonal dissemination of KPC-2- and NDM-1-coharboring Citrobacter freundii in China with an IncR plasmid
Source: Microbiol Spectr. 2024 Dec 19;13(2):e01953-24. doi: 10.1128/spectrum.01953-24 (PMC11792461; doi:10.1128/spectrum.01953-24)
Supplement: Table S2 — The direct repeat sequences of the region of blaKPC-2 and blaNDM-1 in pC275-2. [file spectrum.01953-24-s0002.doc]

Table S2 The direct repeat sequences of the region of *bla*_KPC-2_ and *bla*_NDM-1_ in pC275-2

| MGEs | IRs | Start | End | Sequence (5'→3') |
| --- | --- | --- | --- | --- |
| ISK*pn27* | DR | 24642 | 24647 | TATAGG |
|  | DR | 25728 | 25733 | TATAGG |
| ISK*pn6* | DR | 27021 | 27022 | TA |
