# Supplementary material for: Emergence and clonal dissemination of KPC-2- and NDM-1-coharboring Citrobacter freundii in China with an IncR plasmid
Source: Microbiol Spectr. 2024 Dec 19;13(2):e01953-24. doi: 10.1128/spectrum.01953-24 (PMC11792461; doi:10.1128/spectrum.01953-24)
Supplement: Table S3 — Genetic context of blaNDM-1 on pC275-2，pHNCF44W_NDM-1，pCR-13-12-NDM-1 and pA1705-NDM. [file spectrum.01953-24-s0003.doc]

Table S3 Genetic context of *bla*_NDM-1_ on pC275-2，pHNCF44W_NDM-1，pCR-13-12-NDM-1 and pA1705-NDM

| **Plasmid** | **Start** | **End** | **Direction** | **Gene** | **Annotation** |
| --- | --- | --- | --- | --- | --- |
| pC275-2 | 15818 | 16201 | + | IS*Aba125* | IS30 family transposase |
| pC275-2 | 16302 | 17114 | + | *bla*_NDM-1_ | subclass B1 metallo-beta-lactamase NDM-1 |
| pC275-2 | 17118 | 17483 | + | *ble*-MBL | bleomycin binding protein Ble-MBL |
| pC275-2 | 17488 | 18126 | + | *trpF* | phosphoribosylanthranilate isomerase |
| pC275-2 | 18137 | 19009 | - | *dsbD* | protein-disulfide reductase DsbD family protein |
| pC275-2 | 19205 | 20746 | - | IS*CR1* | IS*91* family transposase |
| pHNCF44W_NDM-1 | 42434 | 43975 | + | IS*CR1* | IS*91* family transposase |
| pHNCF44W_NDM-1 | 44171 | 44470 | + |  | hypothetical protein |
| pHNCF44W_NDM-1 | 44486 | 45043 | + | *dsbD* | twin-arginine translocation (TAT) pathway signal sequence domain protein |
| pHNCF44W_NDM-1 | 45054 | 45692 | - | *trpF* | phosphoribosylanthranilate isomerase |
| pHNCF44W_NDM-1 | 45697 | 46062 | - | *ble*-MBL | bleomycin binding protein Ble-MBL |
| pHNCF44W_NDM-1 | 46066 | 46878 | - | *bla*_NDM-1_ | subclass B1 metallo-beta-lactamase NDM-1 |
| pHNCF44W_NDM-1 | 46979 | 47362 | - | IS*Aba125* | IS30 family transposase |
| pCR-13-12-NDM-1 | 16724 | 17092 | + | IS*Aba125* | Mobile element protein |
| pCR-13-12-NDM-1 | 17193 | 18005 | + | *bla*_NDM-1_ | Subclass B1 beta-lactamase |
| pCR-13-12-NDM-1 | 18009 | 18374 | + | *ble-*MBL | bleomycin resistance protein |
| pCR-13-12-NDM-1 | 18379 | 19017 | + | *trpF* | Phosphoribosylanthranilate isomerase |
| pCR-13-12-NDM-1 | 19028 | 19885 | - | *dsbD* | Oxidoreductase, Gfo/Idh/MocA family |
| pCR-13-12-NDM-1 | 20096 | 21637 | - | IS*CR1* | hypothetical protein |
| pA1705-NDM | 202070 | 202438 | + | IS*Aba125* | Mobile element protein |
| pA1705-NDM | 202539 | 203351 | + | *bla*_NDM-1_ | subclass B1 metallo-beta-lactamase NDM-1 |
| pA1705-NDM | 203355 | 203720 | + | *ble*-MBL | bleomycin resistance protein |
| pA1705-NDM | 203725 | 204363 | + | *trpF* | Phosphoribosylanthranilate isomerase |
| pA1705-NDM | 204374 | 205231 | - | *dsbD* | Oxidoreductase, Gfo/Idh/MocA family |
| pA1705-NDM | 205442 | 206983 | - | IS*CR1* | hypothetical protein |
