# Supplementary material for: Emergence and clonal dissemination of KPC-2- and NDM-1-coharboring Citrobacter freundii in China with an IncR plasmid
Source: Microbiol Spectr. 2024 Dec 19;13(2):e01953-24. doi: 10.1128/spectrum.01953-24 (PMC11792461; doi:10.1128/spectrum.01953-24)
Supplement: Table S4 — Genetic context of blaKPC-2 on pC275-2，pKP048，pA1732-KPC and pKPHS2. [file spectrum.01953-24-s0004.doc]

Table S4 Genetic context of *bla*_KPC-2_ on pC275-2，pKP048，pA1732-KPC and pKPHS2

| **Plasmid** | **Start** | **End** | **Direction** | **Gene** | **Annotation** |
| --- | --- | --- | --- | --- | --- |
| pKP048 | 11831 | 14797 | - | Tn*As1*-*tnpA* | Tn*1721* transposase |
|  | 14801 | 15361 | - | Tn*As1*-*tnpR* | Tn*1721* resolvase |
|  | 15810 | 16370 | + | *repA* | replication protein |
|  | 16904 | 17182 | + |  | hypothetical protein |
|  | 17293 | 17718 | + | *klcA* | antirestriction protein *Klca* |
|  | 17846 | 18001 | + |  | hypothetical protein |
|  | 18047 | 18343 | + | *korC* | transcriptional repressor protein *KorC* |
|  | 18348 | 19328 | + | ISK*pn6*-*tnpA* | putative ISK*pn6*-like transposase |
|  | 19578 | 20459 | - | *bla*_KPC-2_ | carbapenemase KPC-2 |
|  | 20735 | 21715 | - | ISK*pn27*-*tnpA* | putative ISKpn8 transposase |
|  | 21838 | 22395 | - | Tn*2*-*tnpR* | Tn*3* resolvase |
|  | 22558 | 25563 | + | Tn*2*-*tnpA* | Tn*3* transposase |
| pC275-2 | 20904 | 23909 | - | Tn*2*-*tnpA* | Tn*3* family transposase |
|  | 24072 | 24629 | + | Tn*2*-*tnpR* | recombinase family protein |
|  | 24752 | 25732 | + | ISK*pn27*-*tnpA* | IS*481*-like element ISK*pn27* family transposase |
|  | 26008 | 26889 | + | blaKPC-2 | carbapenem-hydrolyzing class A beta-lactamase KPC-2 |
|  | 27139 | 28119 | - | ISK*pn6*-*tnpA* | putative transposase |
|  | 28124 | 28420 | - | *korC* | transcriptional repressor protein KorC |
|  | 28466 | 28621 | - |  | hypothetical protein GR306_29900 |
|  | 28749 | 29174 | - | *klcA* | antirestriction protein |
|  | 29363 | 29608 | - |  | hypothetical protein |
|  | 30059 | 30619 | - | *repA* | replication protein |
|  | 31068 | 31628 | + | Tn*As1*-*tnpR* | recombinase family protein |
|  | 31632 | 34598 | + | Tn*As1*-*tnpA* | Tn***3***-like element TnAs1 family transposase |
|  | 34595 | 34864 | - | IS*903B* | transposase |
|  | 35446 | 35832 | + | *stbA* | hypothetical protein E5AUHO_45010 |
|  | 35972 | 36940 | - | IS*903B* | IS*5* family transposase |
|  | 38343 | 38651 | - | *repB* | replication initiation protein |
|  | 38661 | 39038 | - | IS*1X3*-*tnpA* | IS*1*-like element IS*1X3* family transposase |
|  | 39510 | 40520 | - | *ltrA* | group II intron reverse transcriptase/maturase |
|  | 40632 | 40994 | - |  | maturase |
|  | 41313 | 41834 | + | *umuD* | protein impA |
|  | 41834 | 43105 | + |  | Y-family DNA polymerase |
|  | 43187 | 44161 | - | *parB* | ParB/RepB/Spo0J family partition protein |
|  | 44161 | 45366 | - | *parA* | chromosome partitioning protein ParA |
|  | 45781 | 46050 | + |  | hypothetical protein |
| pA1732-KPC | 208 | 1413 | + | *parA* | AAA family ATPase |
|  | 1413 | 2387 | + | *parB* | ParB family protein |
|  | 2469 | 3740 | - |  | Y-family DNA polymerase |
|  | 3740 | 4171 | - | *umuD* | translesion error-prone DNA polymerase V autoproteolytic subunit |
|  | 4577 | 6063 | + | *ltrA* | group II intron reverse transcriptase/maturase |
|  | 6215 | 6912 | + | IS*1X3*-*tnpA* | IS*1*-like element IS*1X3* family transposase |
|  | 6922 | 7230 | + | *repB* | replication initiation protein |
|  | 8633 | 9601 | + | *IS903B* | IS*5* family transposase |
|  | 9741 | 10127 | - | *stbA* | plasmid stabilization protein StbA |
|  | 10709 | 10942 | + | IS*903B* | transposase |
|  | 10975 | 13939 | - | Tn*As1*-*tnpA* | Tn*3*-like element Tn*As1* family transposase |
|  | 13943 | 14503 | - | Tn*As1*-*tnpR* | recombinase family protein |
|  | 14895 | 15512 | + | *repA* | replication protein |
|  | 16046 | 16324 | + |  | hypothetical protein |
|  | 16435 | 16860 | + | *klcA* | antirestriction protein |
|  | 16988 | 17143 | + |  | hypothetical protein |
|  | 17189 | 17485 | + | *korC* | transcriptional regulator |
|  | 17589 | 18470 | + | ISK*pn6*-*tnpA* | IS*1182*-like element ISK*pn6* family transposase |
|  | 18720 | 19601 | - | *bla*_KPC-3_ | carbapenem-hydrolyzing class A beta-lactamase KPC-3 |
|  | 19877 | 20857 | - | ISK*pn27*-*tnpA* | IS*481*-like element ISK*pn27* family transposase |
|  | 20980 | 21441 | - | Tn*3* | recombinase family protein |
|  | 21493 | 22195 | - | IS*26* | IS*6*-like element IS*26* family transposase |
| pKPHS2 | 17943 | 18665 | + | IS*26* | hypothetical protein |
|  | 18705 | 19178 | + | Tn*3* | resolvase |
|  | 19301 | 20281 | + | ISK*pn27*-*tnpA* | putative transposase |
|  | 20557 | 21438 | + | *bla*_KPC-2_ | Class A Carbapenemase KPC-2 |
|  | 21688 | 22668 | - | ISK*pn6*-*tnpA* | putative transposase |
|  | 22673 | 22969 | - | *korC* | transcriptional repressor protein KorC |
|  | 23015 | 23170 | - |  | hypothetical protein |
|  | 23298 | 23723 | - | *klcA* | antirestriction protein Klca |
|  | 23834 | 24112 | - |  | hypothetical protein |
|  | 24646 | 25206 | - | *repA* | putative replication protein |
|  | 25655 | 26215 | + | Tn*As1*-*tnpR* | Tn4653 resolvase |
|  | 26219 | 29185 | + | Tn*As1*-*tnpA* | transposase for transposon Tn*1721* |
|  | 29327 | 29521 | + | IS*26* | transposase IS*26* |
