# Supplementary material for: Emergence and clonal dissemination of KPC-2- and NDM-1-coharboring Citrobacter freundii in China with an IncR plasmid
Source: Microbiol Spectr. 2024 Dec 19;13(2):e01953-24. doi: 10.1128/spectrum.01953-24 (PMC11792461; doi:10.1128/spectrum.01953-24)
Supplement: Table S5 — Characteristics of all carbapenem-resistant Citrobacter freundiis included in comparative genomic analysis. [file spectrum.01953-24-s0005.doc]

Table S5. Characteristics of all carbapenem-resistant *Citrobacter freundii*s included in comparative genomic analysis.

| Assembly | Collection date | strain | Location | Isolation source | host | ST | ORG |
| --- | --- | --- | --- | --- | --- | --- | --- |
| GCA_905232515 | 2021 | AI2661 | Spain | / | Human-derived | 22 | *bla*_OXA-48_ |
| GCA_032818495 | 2022 | BC73 | China | urine | Human-derived | 22 | *bla*_IMP-4_, *bla*_NDM-1_ |
| GCA_023658085 | 2019 | 59174 | USA | / | Human-derived | 22 | *bla*_KPC-31_ |
| GCA_905329885 | 2021 | AI2826 | Spain | / | Human-derived | 22 | *bla*_KPC-2_ |
| GCA_020809005 | 2020 | DY2010 | China | urine | Human-derived | 22 | *bla*_NDM-5_ |
| GCA_030064365 | 2020 | Cf-Emp | Italy | Rectal swab | Human-derived | 22 | *bla*_KPC-2_, *bla*_OXA-181_, *bla*_VIM-1_ |
| GCA_014170055 | 2018 | IDR1800045912-01-00 | USA | urine | Human-derived | 22 | *bla*_KPC-2_ |
| GCA_900520335 | 2018 | E2614 | Germany | unkown | Human-derived | 22 | *bla*_VIM-1_ |
| GCA_014725915 | 2016 | N16-03880 | Canada | trachea | Human-derived | 22 | *bla*_OXA-204_ |
| GCA_014189355 | 2018 | N18-04078 | Canada | / | Human-derived | 22 | *bla*_OXA-204_ |
| GCA_014189285 | 2018 | N18-04128 | Canada | Rectu | Human-derived | 22 | *bla*_OXA-204_ |
| GCA_014189375 | 2018 | N18-04085 | Canada | Rectum | Human-derived | 22 | *bla*_OXA-204_ |
| GCA_001022275 | 2015 | CAV1741 | USA | respiratory | Human-derived | 22 | *bla*_KPC-2_ |
| GCA_001022155 | 2010 | CAV1321 | USA | Urine/Genitourinary | Human-derived | 22 | *bla*_KPC-2_ |
| GCA_023612475 | 2019 | MEI002 | Switzerland | environment | environmental | 22 | *bla*_OXA-48_ |
| GCA_030007795 | 2014 | CF13 | Spain | gut | Human-derived | 22 | *bla*_OXA-48_ |
| GCA_030007575 | 2014 | CF12 | Spain | gut | Human-derived | 22 | *bla*_OXA-48_ |
| GCA_905232395 | 2021 | AI2609 | Spain | / | Human-derived | new1 | *bla*_VIM-1_ |
| GCA_026240835 | 2018 | CF1807 | China | urine | Human-derived | 107 | *bla*_KPC-2_, *bla*_NDM-1_ |
| GCA_013371705 | 2018 | ZY198 | China | urine | Human-derived | 19 | *bla*_NDM-1_ |
| GCA_032346775 | 2019 | isolateG | Canada | / | Human-derived | 18 | *bla*_NDM-1_ |
| GCA_010365585 | 2019 | 62 | Switzerland | environment | environmental | 632 | *bla*_KPC-2_ |
| GCA_022430505 | 2019 | SCLZS47 | China | environment | environmental | 116 | *bla*_KPC-2_, *bla*_NDM-1_ |
| GCA_900520375 | 2018 | U2785 | Germany | unkown | Human-derived | 116 |  |
| GCA_033164915 | 2019 | F3419 | China | urine | Human-derived | 116 | *bla*_NDM-5_ |
| GCA_030177735 | 2023 | CRNMS1 | China | Patients | Human-derived | 116 | *bla*_NDM-5_ |
| GCA_905338045 | 2021 | AI3057 | Spain | / | Human-derived | 514 | *bla*_KPC-2_ |
| GCA_016939615 | 2019 | CF49969 | Czech Republic | urine | Human-derived | 8 | *bla*_KPC-2_ |
| GCA_905219065 | 2021 | AI2642 | Spain | / | Human-derived | 8 | *bla*_IMP-22_, *bla*_KPC-2_ |
| GCA_008931505 | 2012 | E33 | Australia | environment | environmental | 8 | *bla*_IMP-4_ |
| GCA_008931485 | 2014 | C50 | Australia | clinical sample | Human-derived | 8 | *bla*_IMP-4_ |
| GCA_017086505 | 2019 | zone4 | Switzerland | environment | environmental | 505 | *bla*_OXA-48_ |
| C2036 | 2020 | C2036 | China | urine | Human-derived | 523 | *bla*_KPC-2_, *bla*_NDM-1_ |
| C2085 | 2020 | C2085 | China | urine | Human-derived | 523 | *bla*_KPC-2_, *bla*_NDM-1_ |
| C2002 | 2020 | C2002 | China | urine | Human-derived | 523 | *bla*_KPC-2_, *bla*_NDM-1_ |
| C2028 | 2020 | C2028 | China | urine | Human-derived | 523 | *bla*_KPC-2_, *bla*_NDM-1_ |
| C2024 | 2020 | C2024 | China | urine | Human-derived | 523 | *bla*_KPC-2_, *bla*_NDM-1_ |
| C2084 | 2020 | C2084 | China | urine | Human-derived | 523 | *bla*_KPC-2_, *bla*_NDM-1_ |
| C2177 | 2020 | C2177 | China | urine | Human-derived | 523 | *bla*_KPC-2_, *bla*_NDM-1_ |
| GCA_030122495 | 2020 | 2075 | China | / | Human-derived | 523 | *bla*_KPC-2_, *bla*_NDM-1_ |
| C2075 | 2020 | C2075 | China | urine | Human-derived | 523 | *bla*_KPC-2_, *bla*_NDM-1_ |
| GCA_905218895 | 2021 | AI2602 | Spain | / | Human-derived | 604 | *bla*_KPC-2_ |
| GCA_033157475 | 2019 | F2119 | China | urine | Human-derived | 125 | *bla*_IMP-1_ |
| GCA_014169975 | 2019 | IDR1900015725-01-02 | USA | Rectal swab | Human-derived | 125 | *bla*_KPC-2_ |
| GCA_009857035 | 2018 | L75 | China | urine | Human-derived | 396 | *bla*_NDM-1_ |
| GCA_033162755 | 2021 | F3121 | China | urine | Human-derived | 396 | *bla*_IMP-4_, *bla*_NDM-1_ |
| GCA_033160275 | 2021 | F3021 | China | stool | Human-derived | 396 | *bla*_IMP-4_, *bla*_NDM-1_ |
| GCA_023203275 | 2022 | 2022CK-00286 | USA | Rectal Swab | Human-derived | 611 | *bla*_VIM-1_ |
| GCA_032347795 | 2020 | isolateL | Canada | / | Human-derived | 657 | *bla*_KPC-3_ |
| GCA_023330605 | 2021 | GMU8049 | China | unkown | Human-derived | 257 | *bla*_NDM-1_ |
| GCA_002215385 | 2015 | 45309 | China | environment | environmental | 257 | *bla*_KPC-2_ |
| GCA_030178515 | 2018 | 2017-45-172 | USA | swab | Human-derived | 311 | *bla*_KPC-3_ |
| GCA_030412665 | 2018 | 2017-45-137-03 | USA | environment | environmental | 341 | *bla*_KPC-3_ |
| GCA_032345935 | 2019 | isolateH | Canada | / | Human-derived | 98 | *bla*_OXA-48_ |
| GCA_024662035 | 2021 | CF10 | China | / | Human-derived | 969 | *bla*_KPC-2_, *bla*_NDM-1_ |
| GCA_030166475 | 2022 | THB2 | China | environment | environmental | 503 |  |
| GCA_013740875 | 2017 | RHBSTW-00444 | United Kingdom | environment | environmental | unkown |  |
| GCA_018128425 | 2019 | 3347689II | Switzerland | outer malleolus | Human-derived | unkown | *bla*_NDM-1_ |
| GCA_019803045 | 2017 | CCRI-24236 | Canada | anal swab | Human-derived | 686 | *bla*_KPC-2_ |
| GCA_015353115 | 2013 | CA71 | China | / | Human-derived | 716 | *bla*_IMP-4_ |
| GCA_905337845 | 2021 | AN2351 | Spain | / | Human-derived | 985 | *bla*_OXA-48_ |
| GCA_032945095 | 2018 | K1818 | China | sputum | Human-derived | 1118 | *bla*_KPC-2_ |
| GCA_032939275 | 2021 | K1721 | China | stool | Human-derived | 753 | *bla*_KPC-2_ |
| GCA_029025745 | 2020 | L2395 | China | / | Human-derived | unkown | *bla*_NDM-1_ |
| GCA_032947135 | / | K2721 | China | / | Human-derived | new2 | *bla*_KPC-2_ |
| GCA_032946055 | / | K2521 | China | / | Human-derived | new2 | *bla*_KPC-2_ |
| GCA_905337805 | 2021 | AN2353 | Spain | / | Human-derived | unkown | *bla*_OXA-48_ |
| GCA_032938575 | 2018 | K1018 | China | throat swab | Human-derived | 690 | *bla*_KPC-2_ |
| GCA_032944275 | 2018 | K1118 | China | sputum | Human-derived | 690 | *bla*_KPC-2_ |
| GCA_032943435 | / | K616 | China | / | Human-derived | 690 | *bla*_KPC-2_ |
| GCA_032940055 | 2022 | K4022 | China | urine | Human-derived | 690 | *bla*_KPC-2_ |
| GCA_033153635 | 2019 | K1219 | China | sputum | Human-derived | 690 | *bla*_KPC-2_ |
| GCA_032941735 | 2018 | K118 | China | sputum | Human-derived | 690 | *bla*_KPC-2_ |

/: Not available.

new: New ST type

Unknown:Its ST type was not predicted
